# Modified Ultrafast Papanicolaou Stain in Ultrasound Guided FNAC of Intra-abdominal Lesions

**DOI:** 10.30699/ijp.2020.98405.1971

**Published:** 2020-02-26

**Authors:** Sweta Kamalkant Shastri, Archana Joshi

**Affiliations:** Department of Pathology, N.K.P Salve Institute of Medical Sciences and Research Centre, Nagpur, Maharashtra, India

**Keywords:** Fine needle aspiration cytology, Intra-abdominal lesions, Modified Ultrafast Papanicolaou Stain, Papanicolaou Stain, Quality Index

## Abstract

**Background & Objective:**

Modified Ultra-fast Papanicolaou (MUFP) stain has been developed from Papanicolaou stain (PAP) with the goal to improve staining quality, minimize staining time for obtaining immediate cytological diagnosis and to check specimen adequacy during Ultrasound guided Fine needle Aspiration Cytology (US guided FNAC). The aim of this research was to study the cytomorphological features of intra-abdominal lesions with help of US guided FNAC and to assess the diagnostic utility of Modified Ultrafast Papanicolaou stain in cytological diagnosis.

**Methods:**

This cross-sectional study enrolled consecutive 100 subjects in N.K.P Salve Institute of Medical Sciences and Research Centre, Nagpur, which is a tertiary teaching hospital in India, from July 2015 to June 2017 who underwent US guided FNAC for Intra-abdominal lesions. Fine needle aspiration was done under ultrasound guidance and the smears were divided into two groups. Wet smears were fixed in 95% ethyl alcohol for conventional PAP staining and air dried for MUFP. After staining, results were evaluated on basis of the cytological features. Scores were given according to four parameters namely background of smears, staining pattern, cell morphology and nuclear staining. Quality index was calculated from the ratio of score achieved to the possible maximum score.

**Results:**

The most common organs involved were ovaries (46 %) followed by liver (11%) and most common lesions were malignant (68 %). The cytological characteristic showed significant difference in all four parameters (*P*<0.05) when MUFP stain smears were compared with PAP stain smears. There was also statistically significant difference when cumulative score and Quality Index were compared (*P*<0.001) between the two stains.

**Conclusion:**

The US guided Fine needle aspiration (FNA) is simple, safe, rapid and inexpensive technique useful in cytological diagnosis. MUFP stain is fast, reliable and has better diagnostic utility for cytological diagnosis when compared to PAP stain.

## Introduction

Evaluation of deep non-palpable mass or focal lesions involving intra-abdominal sites is often difficult (1). Introduction of diagnostic imaging techniques like ultrasonography (US) has enabled the detection and location of these lesion, but imaging techniques does not always distinguish between malignant and benign lesion. A correct tissue diagnosis is essential for treatment and staging of malignancy (2).

Fine needle aspiration cytology (FNAC) is a well-established technique and is gaining popularity in diagnosing intra-abdominal lesion. (3,4) With the use of radiological guidance for needle placement, this technique is an effective way to obtain diagnostic material of deep seated intra-abdominal lesion for rapid and accurate diagnosis (2,5-7). FNAC is a rapid, economical, simple, inexpensive and safe diagnostic procedure without radiation hazards. This procedure helps in decreasing use of hospital resources, reduces patient discomfort, and morbidity (8-10).

The need of minimal turnaround time for assessing fine needle aspirate smear lead to innovation in staining procedure that required less staining time. Modifications have been developed in Papanicolaou stain (PAP) to improve staining quality and minimize staining time (11,12). Modified Ultra-fast Papanicolaou (MUFP) stain has been introduced for obtaining immediate cytological diagnosis and to check specimen adequacy during radiologically guided FNAC (13). In MUFP, fixation is not required and the staining time is 130 seconds, thus it is very useful for rapid assessment of adequacy of sample, for rapid diagnosis and also helpful in cases where repeating procedure is required in same setting if inadequate sample is obtained during aspiration, thus making it cost effective for both patient and hospital (11). 

The present study was planned for categorizing, studying the cytomorphological features of intra-abdominal lesions with the help of US guided FNAC and to assess the diagnostic utility of MUFP stain in immediate cytological diagnosis.

## Materials and Methods

This cross-sectional study was conducted in N.K.P Salve Institute of Medical Sciences and Research Center, Nagpur, which is a tertiary teaching hospital of India, from July 2015 to June 2017. Consecutive patients who underwent ultrasound guided FNAC for intra-abdominal lesion during study period were screened for eligibility. Written informed consent was taken from the patients before enrolment in the study in a pre-designed proforma. The study was approved by institutional research board (IRB) of the college.

Inclusion criterion was: Patient with intra-abdominal lesion undergoing US guided FNAC with adequate material for making slides. Exclusion criteria included conditions such as consent not given by patient and acellular aspirate on FNAC.

During the study period 110 patients were screened, out of them 10 patients were excluded as the aspirate was acellular. The clinical and radiological details of the patients were noted in pre-designed patient proforma. Under US guidance fine needle aspiration (FNA) was done using 20 mL disposable syringe having 23 gauze needle. Lumbar puncture needle of the same thickness was used for deep seated lumps, which was fitted with a 10 mL syringe. Then, 5-6 smears were prepared from the aspirated material. The smears were divided into two groups, wet smears were fixed in 95% ethyl alcohol for PAP stain and air dried for MUFP stain. The rest of staining steps for PAP and MUFP stains were done as described in Table 1.

After staining, results were evaluated on basis of cytological feature. Scores were given according to four parameters namely Background of smears, Overall Staining, Cell morphology and Nuclear characteristics. Quality index was calculated from the ratio of score achieved to the maximum score possible (maximum possible score was 11).

**Sample Size Calculation:**

Hospital data of last five year were screened to see the number of patients visiting the hospital with abdominal mass and then sample size of 100 was taken into account for the study time period.

**Statistical Analysis**

All the data was entered in Microsoft office excel sheet and then analysed. SPSS 20 (SPSS Inc., Chicago, Ill. USA) was used in statistical analysis. For continuous data, Mean ± Standard deviation was derived. For paired data analysis “Student Paired T-test” was used and P-value< 0.05 was considered statistically significant. Statistical analysis was done both separately and cumulatively for all components of stain characteristics defined in the study protocol.

**Table 1:** Comparing the Modified Ultrafast Papanicolaou (MUFP) and Papanicolaou (PAP) staining on the basis of staining steps and procedure time

MUFP	Routine PAP
Staining Procedure	
Air Dried smears kept in Normal saline for 30 sec and then in alcoholic formalin for 10 sec.	Smears wet fixed for 30 min 70% ethanol – 1min, 50% ethanol –1min
Tap water 6 slow dips	Distilled water 6 dips
Hematoxylin 30 second	Harris Haematoxylin 5 minutes
Tap water 6 slow dips	Rinse in tap water for 2 minutes
Isopropyl alcohol 95% 6 dips	Rinse in Scott’s tap water for 2 minutes
Eosin Alcohol-36 (EA 36) for 15 seconds	Rinse in tap water for 2 minutes
Isopropyl alcohol 95% 6 dips	Ethanol 70% for 2 minutes
Isopropyl alcohol 100% 6 dips	Ethanol 95% for 2 minutes
Xylene 10 slow dips	Ethanol 95% for 2 minutes
Distyrene Plasticizer Xylene (DPX)	OG-6 for 2 minutes
Mount with cover slip	Rinse in 95% Ethanol two changes 2 minutes each
	EA 50 for 2 minutes
	Rinse in 95% Ethanol for 1 minute
	Air dry
	Xylene minutes
	DPX and mount with cover slip
Procedure time	
Total staining time-130 secs 10 second fixation (air dry)	Total staining time-15 mins 30 minutes fixation (wet dry)

## Results

In this study, out of 110 patients 100 fulfilled the inclusion criteria and 10 patients were excluded as slides showed no cellularity. Thus, 100 cases who underwent US guided FNAC of intra-abdominal lesions followed by rapid staining by MUFP and routine staining for cytological diagnosis were studied. 

**Fig. 1:**
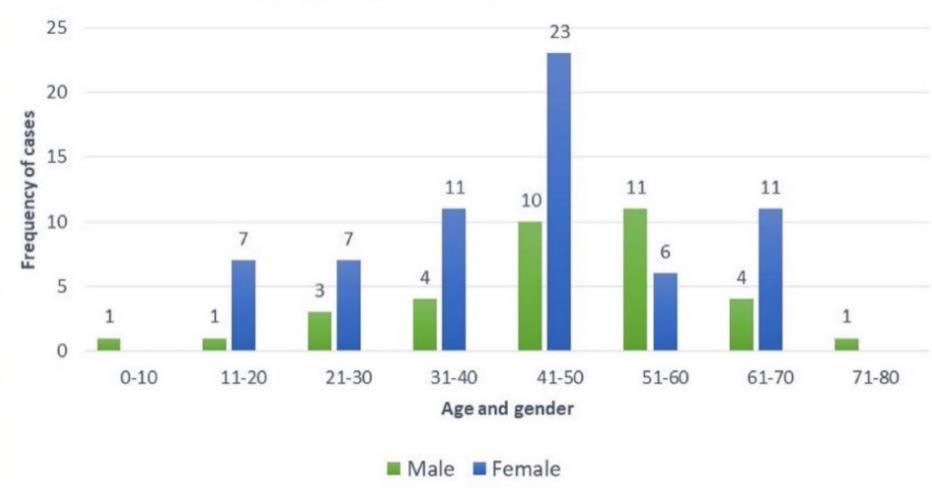
Distribution of cases according to age and gender

**Fig. 2:**
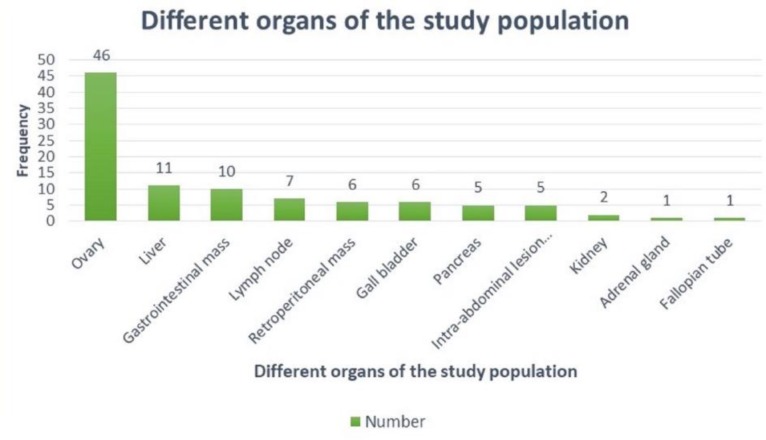
Distribution of cases according to different organs involved in the study population

**Fig. 3:**
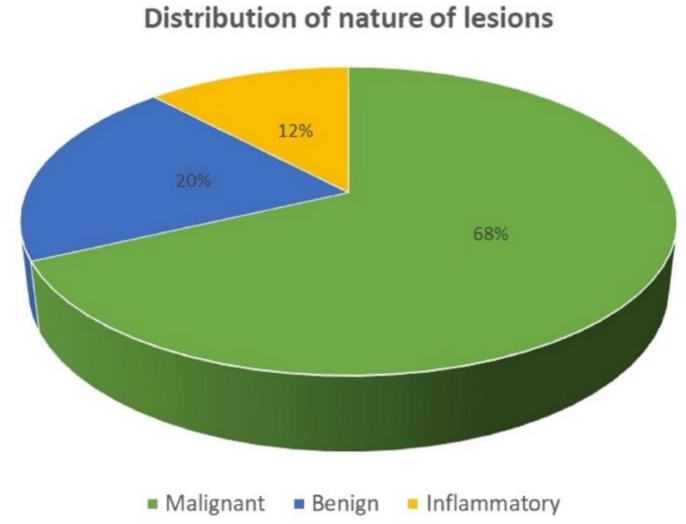
Distribution of nature of cases according to cytological diagnosis

The age of the patients ranged from 6 years to 77 years with mean age being 44.65±15.12 years with 65% being female gender (Figure 1). The most two common involved organs in the study were ovary (46%) and liver (11%) (Figure 2). There were 68% malignant lesions followed by 20% of being benign in nature and rest were inflammatory (Figure 3). The most common clinical presentations of patients in decreasing order were abdominal mass (70%), anorexia (65%), abdominal pain (58%), diarrhoea (46%), constipation (35%), fever (32%), weight loss (25%), hepatomegaly (6%), and jaundice (5%).

The most common malignant neoplasm in ovary was serous cystadenocarcinoma (15%), followed by mucinous cystadenocarcinoma (5%). The most common benign neoplasm in ovary was benign mucinous cystadenoma, whereas inflammatory lesion included non-specific inflammation of ovary. The most common malignant lesions in liver were metastatic (5%) followed by hepatocellular carcinoma (4%), whereas the most common non-malignant lesion was pyogenic abscess 2%. The other malignant lesion diagnosed on US guided FNAC were adenocarcinoma of various organs, sarcoma, neuroendocrine carcinoma, renal cell carcinoma, metastatic carcinoma, non-Hodgkin’s lymphoma, and pheochromocytoma. 

**Fig. 4:**
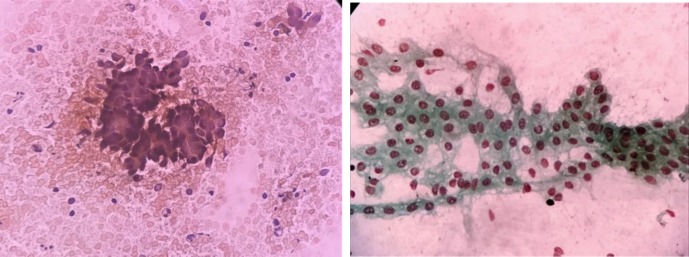
Comparison of MUFP and PAP cytology staining for serous papillary cystadenocarcinoma ovary showing clusters of round to oval cells with hyperchromatic nuclei, moderate anisonucleosis and pleomorphism

**Fig. 5:**
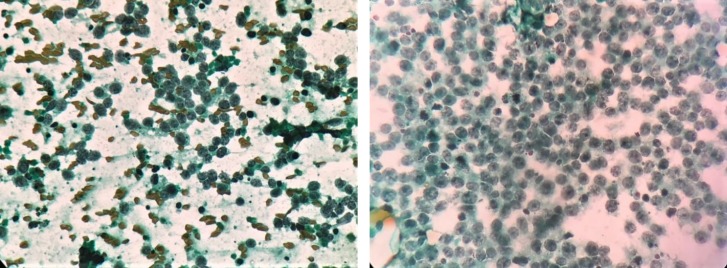
Comparison of MUFP and PAP cytology staining for round cell tumor (intra-abdominal mass unknown origin); (a) Haemorrhagic background with moderately preserved cell morphology, dull nuclear characteristics, and overall moderately good staining pattern (PAP 40x); (b) Clean background with well-preserved cell morphology, crisp nuclear characteristics, and overall good staining pattern (MUFP 40x)

**Fig. 6:**
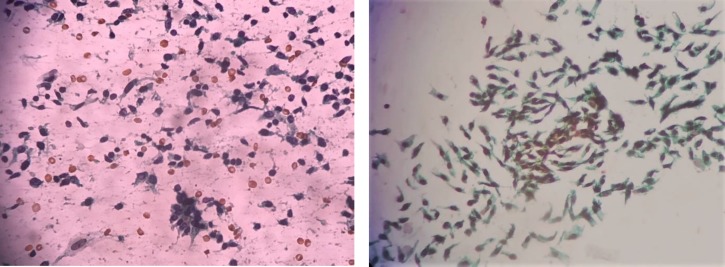
Comparison of MUFP and PAP cytology staining of aspirate from retroperitoneal mass showing spindle cell tumor (a) Haemorrhagic background with moderately preserved cell morphology, dull nuclear characteristics, and overall moderately good staining pattern (PAP 40x) (b) Clean background with well-preserved cell morphology, crisp nuclear characteristics, and overall good staining pattern (MUFP 40x)

When the smears stain with MUFP and PAP stain were compared, smears stained with MUFP showed clean background and overall good cellular morphology when compared with PAP (Figures 4, 5 & 6). The comparison of score and quality index of MUFP and PAP stain showed MUFP stain having better quality index than PAP stain. The cytological characteristic showed significant difference in all four parameters (*P*<0.05) when MUFP stain smears were compared to PAP stain smears (Tables 2 & 3).

The cyto-histopathological correlation was available in only 45 cases because either in patients who were advised for biopsy, consent was not given for surgical intervention or they were lost in follow up. Sensitivity and diagnostic accuracy were calculated for 45 cases (Table 4).

**Table 2:** Table showing various characteristic of the stain used for comparing Modified Ultrafast Papanicolaou (MUFP) and Papanicolaou (PAP) stain

Characteristic of the stain	MUFP Stain n (%)	PAP Stain n (%)
Background		
Clean (Score 2)	100(100)	37(37)
Haemorrhagic (Score 1)	0(0)	63(63)
Overall Staining		
Good (Score 3)	70(70)	50(50)
Moderate (Score 2)	30(30)	50(50)
Bad (Score 1)	0(0)	0(0)
Cell Morphology		
Good (Score 3)	96(96)	89(89)
Moderate (Score 2)	4(4)	11(11)
Bad (Score 1)	0(0)	0(0)
Nuclear Characteristic		
Good (Score 3)	91(91)	78(78)
Moderate (Score 2)	9(9)	22(22)
Bad (Score 1)	0(0)	0(0)

Score 1 = Haemorrhagic background, Overall staining bad, Cell morphology not preserved, Nuclear details not preserved; Score 2 = Clean background, Overall staining moderately good, Cell morphology moderately preserved, Nuclear details moderately preserved; Score 3 = Good overall staining, Cell morphology well preserved and crisp, Nuclear details well preserved and crisp

**Table 3:** Table showing comparison of different staining parameters that are used to study Modified Ultrafast Papanicolaou (MUFP) and Papanicolaou (PAP) stain

Characteristic	MUFP STAIN (Mean ± SD)	PAP STAIN (Mean ± SD)	P-value
Background of smears	2.00 ± 0.00	1.37± 0.48	0.00
Staining pattern	2.68 ± 0.47	2.50 ± 0.50	0.00
Cell morphology	2.95± 0.22	2.85± 0.31	0.11
Nuclear staining	2.91 ± 0.28	2.78± 0.42	0.01
Cumulative score	10.53± 0.52	9.58± 0.62	0.00
Total score of study population (maximum score – 1100)	1053	958	0.00
Quality index	0.95	0.87	0.00

**Table 4:** Cytohistopathological correlation and diagnostic accuracy of various lesions

Lesion	Cytological diagnosis	Histological diagnosis available	Correlation of cytological and histological diagnosis	Diagnostic accuracy
Malignant	68	30	30	100
Benign	20	15	12	80
Inflammatory	12	-	-	
Overall				93.33

(a) Haemorrhagic background is obscuring cellular details (PAP 40 X) (b) Clean background showing papillary pattern (MUFP 40 X)

## Discussion

This was a study on 100 patients who underwent US guided FNAC for abdominal lesions. In the study US guided FNAC was performed followed by rapid staining of aspirated material by MUFP stain. Ovary was the most common organ involved in the study population. The results of the study showed that MUFP has statistically significant better results in rapid assessment of smears when compared to PAP stain. There was also good sensitivity and diagnostic accuracy of US guided FNAC when compared to cyto-histology. The aspirated material were mostly adequate in malignant lesions, as compared to benign and non-neoplastic lesions, suggesting that US guided FNAC should be routinely done in deep seated lesions because of the high adequacy rate and very low complication rate (14).

US guided FNAC is used as a means of diagnosing lesions in intra-abdominal organs (1). It helps in collection of cellular material with high accuracy rate (15). The use of US guidance for needle placement allows aspiration of representative material from specific anatomical site for precise cytological diagnosis. The male to female ratio was 1: 1.8 in this study, which is comparable to study done by Reddy *et al.* (14). Mostly lesions were malignant followed by benign and inflammatory. These findings were similar to studies conducted by Islam *et al. *(1), Ghodasara *et al. *(16), and Hemalatha* et al. *(17), Reyaz *et al. *(18), and Namshiker* et al. *(19). The most common involved organ in the present study was ovary and most common cytological diagnosis in ovary was malignant neoplasm which was similar to study conducted by Bandyopadhyay* et al. *(20) and Pal* et al. *(21). The most common malignant lesion in liver was metastatic deposit secondary to adenocarcinoma carcinoma which was similar to study conducted by Reyaz *et al. *(18) and Namshiker* et al. *(19). 

The routine PAP stain involves wet fixation and subsequent staining, requiring at least 30 minutes. With the motive of decreasing the staining time, the rapid PAP stains were developed with respective staining time of 4 minutes, 5 minutes and 90 seconds (11,22). But the main drawback it had, was the quality of rapid stains, which was not satisfactory and required wet fixation. To overcome these problems, Yang and Alvarez developed Ultra-Fast Pap (UFP) stain which is a hybrid of the technique by Romanowsky and conventional Pap stain, and reduced the staining time to 90 seconds. It was preferably used for thyroid cytology (23). 

Kamal* et al. *(24) from India further modified the Ultra-Fast Pap (UFP) stain (to overcome the problem of shortage of Richard- Allan hematoxylin, Richard- Allan cytostain and ethyl alcohol reagents in the Indian set-up. We adopted Kamal’s MUFP staining for evaluating the FNAC smears of various organs, by replacing Gill’s hematoxylin with the easily available Harris hematoxylin, and compared the results with them. This method required short staining time of 130 seconds and the cytomorphology was well seen. This method will be helpful to achieve rapid cytological diagnosis and will be helpful to know adequacy of aspirated sample for deciding the need for repeat aspiration if needed.

In present study a correct diagnosis was achieved in all 100 cases. The quality of MUFP staining was evaluated on four parameters namely smear background, overall staining pattern, cell morphology, and nuclear characteristics. The quality index score was better in MUFP stain as compared to routine PAP stain and these findings were similar to studies conducted by Alwahibi* et al. *(25) and Sinkar* et al. *(26).

The advantages of MUFP stain when compared to rapid PAP stain are:

1. As fixation is not required, the staining time is 130 seconds and therefore very useful for rapid assessment of adequacy of samples and rapid diagnosis.
2. Staining solution can be prepared from locally available reagents.
3. Replacing Gill’s hematoxylin with Harris hematoxylin does not alter the staining characteristics and gives equally good results.
4. Background is clear and RBC free and thus helps in better interpretation.
5. The technique provides good nuclear and cytoplasmic details as the cells appear large with crisp morphological features.
6. Air drying removes the artefactual changes seen in wet fixed smears due to poor fixation.
7. Cell loss with wet fixation is avoided, and therefore recommended for lipid rich tumours like lipoma

The disadvantages of MUFP stain compared to rapid PAP stain are

1) The method is technique sensitive, therefore complete air drying should be strictly observed as inadequate drying can give suboptimal results. Smears need to be properly prepared as thick smears do not give satisfactory results.

2) Due to the omission of Orange G, interpretation of cytoplasmic keratinization is not possible 

3) Normal saline, Harris hematoxylin and EA-36 should be changed regularly as they are storage sensitive

4) Bipolar single nuclei are not stained properly.

5) Universal standardization of MUFP stain is recommended as locally available solutions may influence the results.

6) The pH of the alcoholic formalin should be maintained at 5.0 or else it can result in poor staining.

## Limitation and Strength

The strength of the study includes large sample size and inclusion of patients with different abdominal organ involvement. The limitation of the study was getting only 45 patient for histo-cytological correlation 

## Conclusion

In this study we concluded that for diagnosis of intra-abdominal lesion, US guided FNAC coupled with rapid staining technique like MUFP will help in getting more satisfactory sample and rapid cytological diagnosis. US guided FNAC is a safe, cost-effective technique for cytological diagnosis of intra-abdominal lesion and MUFP stain helps in rapid diagnosis and also provides information about adequacy of aspirated sample. This will be helpful for patients in getting rapid diagnosis and early treatment and will be cost effective for patients and hospitals.
